# Socio-economic inequality in functional disability and impairments with focus on instrumental activity of daily living: a study on older adults in India

**DOI:** 10.1186/s12889-021-11591-1

**Published:** 2021-08-12

**Authors:** Ratna Patel, Shobhit Srivastava, Pradeep Kumar, Shekhar Chauhan, Mani Deep Govindu, David Jean Simon

**Affiliations:** 1 Department of Public Health and Mortality Studies, International Institute for Population Sciences, Mumbai, India; 2 Department of Mathematical Demography and Statistics, International Institute for Population Sciences, Mumbai, India; 3 Department of Population Policies and Programmes, International Institute for Population Sciences, Mumbai, India; 4 Karnataka Health Promotion Trust, Bangalore, Karnataka India; 5 Paris 1 Pantheon-Sorbonne University, Paris, France

**Keywords:** Functional disability, Impairments, Inequalities, Older adults, India

## Abstract

**Background:**

Studies have examined functional disability among older adults by combining Activities of Daily Living (ADL) and Instrumental Activities of Daily Living (IADL). This study adds another dimension to ADL and IADL by combining various impairments such as hearing, vision, walking, chewing, speaking, and memory loss among older adults. This study examines functional disability among older adults in India as measured by ADL, IADL, along with various impairments.

**Methods:**

This study utilized data from Building a Knowledge Base on Population Aging in India (BKPAI), a national-level survey and conducted across seven states of India. The study utilized three outcome variables, namely, ADL, IADL, and Impairments. Descriptive and bivariate analyses were used along with multivariate analysis to fulfil the objectives of the study. The concentration index was calculated for ADL, IADL, and impairments, and further, decomposition analysis was carried out for IADL.

**Results:**

The results observed that nearly 7.5% of older adults were not fully independent for ADL. More than half (56.8%) were not fully independent for IADL, and nearly three-fourths (72.6%) reported impairments. Overall, ADL, IADL, and impairments were higher among older adult’s aged 80+ years, older adults with poor self-rated health, and those suffering from chronic diseases. The likelihood of ADL (AOR = 6.42, 95% CI: 5.1–8.08), IADL (AOR = 5.08, 95% CI: 4.16–6.21), and impairment (AOR = 3.50, 95% CI: 2.73–4.48) were significantly higher among older adults aged 80+ years compared to 60–69 years. Furthermore, older adults who had poor self-rated health and suffered from chronic diseases were more likely to report ADL (AOR = 2.95, 95% CI: 2.37–3.67 and AOR = 2.70, 95% CI: 2.13–3.43), IADL (AOR = 1.74, 95% CI: 1.57–1.92 and AOR = 1.15, 95% CI: 1.04–1.15), and impairment (AOR = 2.36, 95% CI: 2.11–2.63 and AOR = 2.95, 95% CI: 2.65–3.30), respectively compared to their counterparts. Educational status and wealth explained most of the socio-economic inequality in the prevalence of IADL among older adults.

**Conclusion:**

It is recommended that the government advise older adults to adopt health-promoting approaches, which may be helpful. Further, there is a pressing need to deliver quality care to older adults suffering from chronic conditions.

**Supplementary Information:**

The online version contains supplementary material available at 10.1186/s12889-021-11591-1.

## Background

Dialogues that took place during the World Assembly on Ageing, held in Vienna in 1982, have pushed the focus on ageing around the world [1]. More than 35 years since then, ageing has risen significantly on the policy discourse across the countries [2]. However, the pace at which focus was given on ageing was not similar across the countries, much linked to the countries’ current status of demographic transition [3]. Developed countries have raced ahead of developing countries in providing a healthy and quality life to their older adults [4]. Due to better living conditions and improvements in medicine and technology, life expectancy has increased globally during the last decades [5]. Current demographic projections forecast that the proportion of older people will continue to grow [6]. The segment of older people will grow even faster in developing countries, specifically in India [7]. This prompted us to examine functional disability status among older adults in one of the developing countries, i.e., India. Improving life expectancy and declining fertility has played a significant role in raising the share of older adults in India. Currently, older adults share around 8% of India’s total population, which is expected to rise to 19% by 1950 [8]. The higher share of older adults implies a higher burden of disease and functional disability among older adults [8].

This study intends to examine functional disability among older adults in India. Three different indicators of functional disability were examined in this study: Activity of Daily Living (ADL), Instrumental Activity of Daily Living (IADL), and impairments related to bodily functions like hearing, vision, walking, chewing, speaking, and memory. ADL and IADL have been studied widely to measure functional disability across various settings [9]. However, a limited scholarship is available in examining impairments related to bodily functions along with ADL and IADL [10]. Activities of Daily Living have been categorized into two groups: Basic activities and Instrumental activities [11]. Both ADL and IADL depict functional disability; however, these two are different. Basic ADLs are generally linked to motor functions, whereas Instrumental ADLs are more linked to cognitive functions [12]. In this study, Basic ADL includes bathing, dressing, toilet, mobility, continence, and feeding. Instrumental ADLs encompass activities that are a set of complex voluntary behaviour directed to achieve a goal, such as managing finances, housekeeping, problem-solving, and so on [13]. In this study, IADL includes eight functional limitations: the ability to use a phone, shopping, food preparation, housekeeping, laundry, transportation, medication, and finances.

Activities of Daily Living assume greater relevance in the Indian context as the elderly population rises in India [14]. First proposed by Katz et al. (1963), ADL as an original measure included six activities of daily living, namely, difficulty with bathing, dressing, toileting, transferring, continence, and feeding [15]. This study has the same six activities of ADL, as was proposed by Katz et al. in 1963. The activities included as a measure of ADL in this study have been concordant with the previous studies in the Indian context [16]. Previously available literature in the Indian context noticed that ADL among older adults differs by various socio-economic characteristics [9, 17]. Lawton and Brody (1969) proposed the eight activities as a measure of IADL: using the telephone, managing money, handling medications, preparing meals, doing housework, laundry, transportation, and shopping [18]. This study used the same eight activities to measure the IADL proposed by Lawton and Brody (1969). Various studies have measured functional performance among older adults by self-reported activities of daily living and instrumental activities of daily living; however, these tools do not provide enough data on actual functional capacity among older adults [19]. Therefore, in this study, we have added another dimension and examined various impairments among older adults in India. This study includes six types of impairments: hearing, vision, walking, chewing, speaking, and memory. ADL and IADL precisely measure functional disability; however, impairments measure an actual level of disability among older adults [20].

Extensive research is available on ageing in developed countries; there is a dearth of research on ageing among developing countries like India. There is a lack of epidemiological data from India, and the issue of functional disability along with impairments is one of the issues that has not been given sufficient attention. Therefore, this study aimed to examine the correlates of ADL, IADL, and Impairments among older adults in India. Further, this study examined economic inequality in ADL, IADL, and Impairments among older adults with the concentration curve’s help. Finally, the current study proposes to decompose the socio-economic factors of ADL, IADL, and Impairments.

## Methods

### Data

The research used data from the BKPAI (Building a Knowledge Base on Population Aging in India), a countrywide representative survey conducted in seven Indian states in 2011 [21]. The BKPAI gathered data on a variety of socioeconomic and health issues affecting those aged 60 and up. Kerala, Tamil Nadu, Maharashtra, Himachal Pradesh, Punjab, Odisha, and West Bengal were the seven states chosen for the survey [21]. The sample size was divided evenly across urban and rural locations, regardless of population concentration [21].

About 9852 individual older adults were interviewed from the selected households [21]. The effective sample size for this study was 9541 older adults aged 60 and up from seven states. 311 missing cases were excluded from the present study According to the study’s goal and objective, respondents aged 60 and over were eligible to participate [21].

#### Outcome variables

ADL (Activity of Daily Living), IADL (Instrumental Activity of Daily Living), and Impairments were used as outcome variables in this study. Six questions were asked to the older adults, and the results are in Supplemental file 1. ADLs (Activities of Daily Living) were recoded on a scale of 0 to 6, with a higher score indicating greater independence (Cronbach Alpha: 0.93) [22–24]. A person with a score of 6 was regarded completely independent for ADL, whereas anyone with a score of less than 6 was considered not independent for ADL [25]. A detailed methodology on how we formed ADL is given in Supplementary file 1.

Instrumental daily living activities were graded on a scale of 0 to 8, with a higher score indicating greater independence (For detail, see Supplementary file 1) [25]. A score of 6 or more was recorded as 0, indicating high IADL, while a score of 5 or less was entered as 1, indicating low IADL [25]*.* Anyone with a score of 6 or more was deemed completely independent for IADL, while anyone with a score of less than 6 was considered not independent for IADL.

At last, impairment was coded as 0 means “no impairment,” and 1 means “having an impairment” (For detail, see Supplementary file 1).

#### Predictor variables

Age was coded as (60–69, 70–79, and 80+ years), gender was coded as (men and women), education was coded as (no education, below 5 years, 6–10 years, and 11+ years), marital status was coded as (not in a union and currently in a union), marital status was coded (not in a union and currently in a union), living arrangement was coded as (alone, with spouse, with children and others), working status coded as (no, yes and retired), having children coded as (yes and no), self-rated health coded as (good and poor), chronic disease coded as (no and yes), substance use (no and yes), wealth coded as (poorest, poorer, middle, richer, and richest), religion coded as (Hindu, Muslim, Sikh, and others), Caste coded as (Scheduled Caste (SC), Scheduled Tribe (ST), Other Backward Class (OBC) and others), residence coded as (rural and urban) and states coded as (Himachal Pradesh, Punjab, West Bengal, Orissa, Maharashtra, Kerala, and Tamil Nadu).

Furthermore, the wealth quintile was a significant variable in determining the household’s economic position. In the survey, a household wealth index was constructed by integrating household amenities, assets, and durables and categorising families in a range from poorest to wealthiest, corresponding to wealth quintiles from lowest to highest. For the decomposition analysis, the study utilised a wealth score (continuous variable). The wealth quintile, split into five equal portions, was used to calculate the Concentration Index (CI).

#### Concentration index

For ADL, IADL, and impairments, the concentration index was calculated. The concentration index is derived as twice the weighted covariance between the result and fractional rank in the wealth distribution divided by the variable mean, and it measures the extent of inequality by measuring the area between the concentration curve and line of equality [26].

The concentration index can be written as follows:

$$ \boldsymbol{C}=\frac{\mathbf{2}}{\boldsymbol{\mu}}\boldsymbol{\operatorname{cov}}\left({\boldsymbol{y}}_{\boldsymbol{i},}{\boldsymbol{R}}_{\boldsymbol{i}}\right) $$

Where C represents the concentration index, y i denotes the outcome variable index, R is the fractional rank of person I in the distribution of socio-economic status, cov denotes the covariance, and is the mean of the sample’s outcome variable [27]. The index value ranges from − 1 to + 1 [27, 28].

The study further decomposes the concentration index to better understand the relative contributions of various socioeconomic variables on IADL in older individuals [29]. Because the concentration index result for ADL and impairments did not indicate any observable socio-economic disparity, we only decomposed variables for IADL and not for ADL and impairments [29]. The study employed a regression-based decomposition approach provided by Wagstaff et al. to decompose the socio-economic variables [29].

### Statistical analysis

The preliminary findings were estimated using descriptive statistics and bivariate analysis. During bivariate analysis, the chi-square technique was employed to determine the degree of significance. In addition, binary logistic regression analysis was performed to meet the study’s objectives. The results were reported as an adjusted odd ratio (AOR) with a confidence interval of 95% (CI).

The model is usually put into a more compact form as follows:

$$ \ln \left(\frac{P_i}{1-{P}_i}\right)={\beta}_0+{\beta}_1{x}_1+\dots +{\beta}_M{x}_{m-1}, $$

Where *β*_0_, …. . , *β*_*M*_ are the regression coefficient indicating the relative effect of a particular explanatory variable on the outcome. These coefficients fluctuate depending on the context of the study’s analysis.

## Results

Table 1 depicts the socio-demographic characteristics of older adults. The 60–69-year-old age group had a greater proportion of older adults, half of the older adults had no education, and around 40% of older persons were not members of the union. About 6% of older individuals lived alone, 67% of older adults were unemployed, and 4% of older adults had no children. More than half of the older adults assessed their health as bad, 67% adults had chronic illnesses, and 3% adults used drugs.

**Table 1:** Socio-economic profile of older adults in India

Variable	Sample	Percentage
**Age (years)**		
60–69	5875	61.8
70–79	2606	27.4
80+	1031	10.8
**Sex**		
Men	4517	47.5
Women	4995	52.5
**Educational status**		
No education	4857	51.1
Below 5 years	1948	20.5
6 to 10 Years	2129	22.4
11+ years	578	6.1
**Marital status**		
Not in union	3745	39.4
Currently in union	5767	60.6
**Living arrangement**		
Alone	558	5.9
With spouse	1518	16.0
With children	6696	70.4
Others	740	7.8
**Working status**		
No	6396	67.3
Yes	2307	24.3
Retired	809	8.5
**Having children**		
Yes	9107	95.7
No	405	4.3
**Self-rated health**		
Good	4254	44.7
Poor	5258	55.3
**Chronic diseases**		
No	3356	35.3
Yes	6156	64.7
**Substance use**		
No	6196	65.1
Yes	3316	34.9
**Wealth quintile**		
Poorest	2243	23.6
Poorer	2107	22.2
Middle	1963	20.7
Richer	1766	18.6
Richest	1429	15.0
**Religion**		
Hindu	7549	79.4
Muslim	668	7.0
Sikh	897	9.4
Others	398	4.2
**Caste**		
Scheduled Caste	1976	20.8
Scheduled Tribe	531	5.6
Other Backward Class	3491	36.7
Others	3513	36.9
**Place of residence**		
Rural	7026	73.9
Urban	2486	26.1
**State**		
Himachal Pradesh	1469	15.4
Punjab	1349	14.2
West Bengal	1126	11.8
Orissa	1452	15.3
Maharashtra	1368	14.4
Kerala	1348	14.2
Tamil Nadu	1400	14.7
**Total**	9512	100.0

**Fig. 1:**
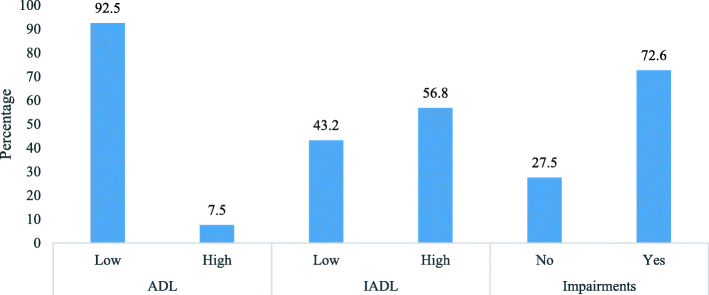
Percentage of older adults suffering from ADL, IADL, and impairments. **Legend:** ADL, IADL, Impairments

The percentage of older adults with ADL, IADL, and impairments is shown in Fig. 1. Only 7% of older adults had a high ADL score, whereas 57 and 73% had high IADL and impairment scores, respectively.

Table 2 shows the percentage distribution of ADL, IADL, and impairment, as well as their adjusted odds ratios, for older individuals by background variables. One-quarter (25.7%) of older individuals aged 80 and above were not totally independent in terms of ADL, whereas 85 and 91% of older adults aged 80 and up suffered from IADL and impairments, respectively. Around 9% of women and 6% of men were not fully independent for ADL, while 74% of women and 71% of men older adults had an impairment. Education exhibited a negative relationship with ADL, IADL, and disability in older individuals. Surprisingly, just 3% of older adults were not totally independent in terms of ADL; nevertheless, IADL and disability affected one-third to two-thirds of older adults living alone. The prevalence of any impairment was greater among older adults who did not work than among those who did. Older adults had poor self-rated health and suffered from chronic diseases were not fully independent for ADL (11.4% & 9.9%), IADL (64.3% & 58.4%), and impairment (83.2% & 82.6%), respectively than their counterparts. For the IADL issue, about 68% of the poorest older adults were not totally independent, whereas 77% of the richest were affected. Although disability was higher in other caste groups, ADL and IADL were more common among lower caste groups (SC/ST). Rural older adults showed higher levels of functional disability and impairment (ADL-7.7%, IADL-59.6%, and impairment-74%) than their urban counterparts.

**Table 2:** Percentage of ADL, IADL, and impairments and their adjusted odds ratio for older adults by background characteristics in India

Variable	ADL		IADL		Impairments	
	%	AOR (95% CI)	%	AOR (95% CI)	%	AOR (95% CI)
**Age (years)**	*		*		*	
60–69^@^	3.4		47.4		65.6	
70–79	9.6	2.33*(1.90,2.86)	66.5	1.85*(1.66,2.07)	81.0	1.86*(1.63,2.11)
80+	25.7	6.42*(5.10,8.08)	85.5	5.08*(4.16,6.21)	90.8	3.5*(2.73,4.48)
**Sex**	*				*	
Men^@^	5.8		56.3		70.7	
Women	9.0	1.02(0.83,1.27)	57.2	0.57*(0.51,0.65)	74.2	1.03(0.90,1.18)
**Educational status**	*		*		*	
None ^@^	9.2		69.7		75.4	
Below 5 years	7.6	1.00(0.80,1.27)	51.2	0.57*(0.50,0.65)	77.0	1.06(0.92,1.24)
6 to 10 years	4.5	0.98(0.74,1.29)	40.5	0.44*(0.38,0.5)	62.0	0.79*(0.68,0.92)
11+ years	3.9	1.48(0.95,2.31)	26.3	0.21*(0.16,0.26)	72.5	1.18(0.93,1.5)
**Marital status**	*		*		*	
Not in union^@^	10.9		63.0		77.4	
Currently in union	5.3	1.01(0.81,1.24)	52.7	0.81*(0.72,0.91)	69.4	0.86*(0.75,0.98)
**Living arrangement**	*		*		*	
Alone^@^	3.4		36.4		66.5	
With spouse	3.6	1.50(0.87,2.57)	48.2	2.79*(2.17,3.59)	62.8	0.78(0.60,1.02)
With children	8.4	2.40*(1.51,3.83)	60.0	4.13*(3.30,5.18)	74.4	0.91(0.72,1.16)
Others	10.1	2.65*(1.57,4.46)	60.4	3.98*(3.04,5.2)	80.5	0.97(0.72,1.30)
**Working status**	*		*		*	
No^@^	10.4		63.3		76.2	
Yes	1.1	0.16*(0.10,0.26)	43.3	0.47*(0.41,0.53)	67.3	0.85*(0.74,0.97)
Retired	2.6	0.41*(0.26,0.63)	43.4	0.81*(0.67,0.97)	58.9	0.83(0.68,1.01)
**Having children**	*				*	
Yes^@^	7.7		57.0		72.7	
No	3.8	0.83(0.50,1.37)	52.0	1.03(0.80,1.32)	69.8	0.86(0.66,1.12)
**Self-rated health**	*		*		*	
Good^@^	2.6		47.5		59.4	
Poor	11.4	2.95*(2.37,3.67)	64.3	1.74*(1.57,1.92)	83.2	2.36*(2.11,2.63)
**Chronic diseases**	*		*		*	
No^@^	3.1		53.9		54.1	
Yes	9.9	2.7*(2.13,3.43)	58.4	1.15*(1.04,1.27)	82.6	2.95*(2.65,3.3)
**Substance use**					*	
No^@^	7.6		56.2		67.9	
Yes	7.3	0.92(0.76,1.12)	57.8	0.92(0.83,1.03)	81.2	1.65*(1.45,1.87)
**Wealth quintile**			*		*	
Poorest^@^	7.9		67.6		76.5	
Poorer	7.4	0.96(0.73,1.26)	59.2	0.88(0.75,1.03)	70.6	0.9(0.76,1.08)
Middle	8.2	0.87(0.65,1.17)	55.3	0.85(0.72,1.01)	70.0	0.86(0.71,1.04)
Richer	6.7	0.67*(0.48,0.92)	51.4	0.74*(0.61,0.89)	69.1	0.98(0.80,1.21)
Richest	7.1	0.72(0.51,1.03)	45.0	0.72*(0.58,0.88)	77.2	1.20(0.94,1.51)
**Religion**	*		*		*	
Hindu^@^	7.3		57.4		71.6	
Muslim	11.7	1.41*(1.03,1.94)	58.1	1.51*(1.23,1.84)	79.1	0.91(0.72,1.14)
Sikh	6.1	1.24(0.79,1.93)	61.3	1.06(0.84,1.34)	71.1	0.78*(0.60,1.00)
Others	6.4	0.85(0.54,1.35)	32.1	0.85(0.66,1.09)	83.8	1.25(0.93,1.69)
**Caste**	*		*			
Scheduled Caste	8.0	1.08(0.85,1.38)	62.9	1.06(0.93,1.22)	74.2	0.92(0.78,1.07)
Scheduled Tribe	5.5	0.86(0.53,1.38)	63.4	0.95(0.75,1.2)	74.4	0.8(0.61,1.04)
Other Backward Class	7.6	1.05(0.83,1.34)	53.4	1.06(0.93,1.21)	65.7	0.83*(0.72,0.96)
Others^@^	7.4		55.7		78.2	
**Place of residence**	*		*		*	
Rural^@^	7.7		59.6		74.1	
Urban	6.8	1.00(0.83,1.21)	48.7	0.85*(0.77,0.95)	68.1	1.06(0.94,1.19)
**State**	*		*		*	
Himachal Pradesh^@^	7.8		60.2		67.9	
Punjab	5.8	0.52*(0.35,0.79)	60.6	1.05(0.85,1.31)	73.3	1.02(0.81,1.28)
West Bengal	11.2	1.19(0.86,1.64)	69.3	1.74*(1.44,2.11)	88.0	2.51*(1.98,3.17)
Orissa	8.5	1.34(0.96,1.87)	71.1	2.21*(1.83,2.67)	82.9	2.62*(2.11,3.24)
Maharashtra	3.9	0.49*(0.33,0.71)	49.3	0.77*(0.65,0.93)	72.9	1.14(0.94,1.38)
Kerala	9.7	0.91(0.64,1.28)	34.5	0.35*(0.29,0.43)	82.2	1.38*(1.1,1.72)
Tamil Nadu	6.0	1.31(0.91,1.89)	53.3	1.25*(1.02,1.52)	44.0	0.51*(0.42,0.63)
**Total**	7.5		56.8		72.6	

*p < 0.05; ^@^Reference category; *ADL* Activities of daily living, *IADL* Instrumental activities of daily living, *AOR* Adjusted odds ratio, *CI* confidence interval; The analysis was adjusted for all the socio-demographic and economic correlates

Results from logistic regression show that the likelihood of ADL (AOR = 6.42, 95% CI: 5.1–8.08), IADL (AOR = 5.08, 95% CI: 4.16–6.21), and impairment (AOR = 3.50, 95% CI: 2.73–4.48) were significantly higher among older adults aged 80+ years compared to 60–69 years. Older adults with 6–10 years of schooling had lower IADL and impairment odds than older adults who were not educated. Older adults living with children were more likely to report ADL (AOR = 2.40, 95% CI: 1.51–3.83) and IADL (AOR = 2.65, 95% CI: 1.57–4.46), related problem respectively, compared to older adults living alone. Working older adults had lower odds of ADL, IADL, and impairment than those who were not working. Older adults who had poor self-rated health and suffered from chronic diseases were more likely to report ADL (AOR = 2.95, 95% CI: 2.37–3.67 and AOR = 2.70, 95% CI: 2.13–3.43), IADL (AOR = 1.74, 95% CI: 1.57–1.92 and AOR = 1.15, 95% CI: 1.04–1.15), and impairment (AOR = 2.36, 95% CI: 2.11–2.63 and AOR = 2.95, 95% CI: 2.65–3.30), respectively compared to their counterparts.

### Results for concentration curve and decomposition analysis

**Fig. 2:**
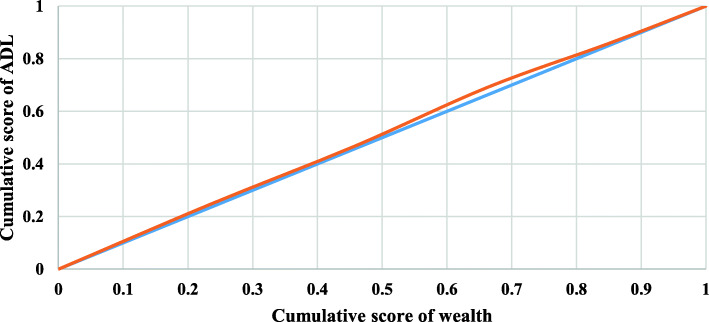
Concentration curve for ADL among older adults in India. **Legend:** Cumulative score of wealth

**Fig. 3:**
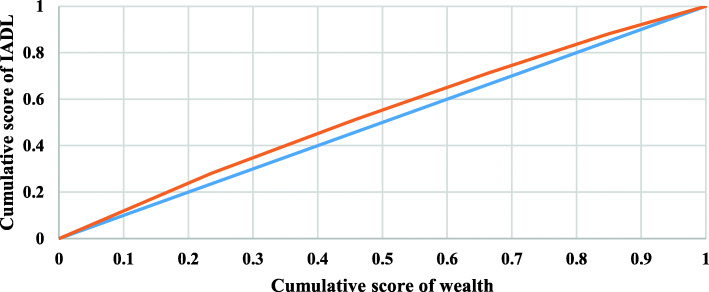
Concentration curve for IADL among older adults in India. **Legend:** Cumulative score of wealth

**Fig. 4:**
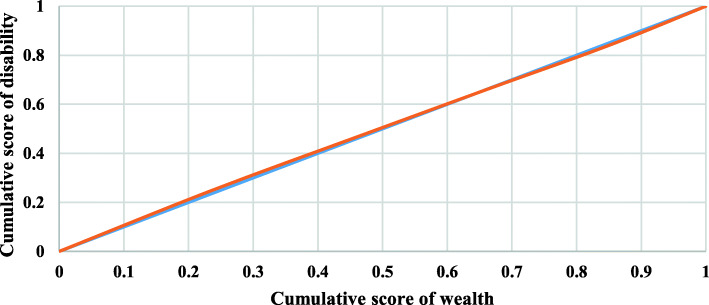
Concentration curve for impairments among older adults in India. **Legend:** Cumulative score of wealth

Concentration curves for ADL, IADL, and impairments among older adults in India were displayed in Figs. 2, 3, and 4, respectively. Inequality is concentrated towards the rich if the curve is created below the line of equality, and vice versa. Furthermore, the bigger the inequality, the larger the region between the line of equality and the curve. The result noticed inequality of − 0.001 (Fig. 2), − 0.073 (Fig. 3) and − 0.0004 (Fig. 4), respectively. The result noticed that inequality was significantly higher for IADL (− 0.073) than ADL (− 0.001) and impairment (− 0.0004). The results noticed negligible inequality for ADL and impairment (There was inequality as the CI is almost 0), so this study could not decompose the factors for these two variables.

Table 3 shows the decomposition analysis estimates for the contribution of several variables for IADL among older adults in India. The product of elasticity and CI is the absolute contribution, whereas the percentage contribution is the proportion of the individual’s absolute contribution. Furthermore, the logistic regression coefficient is referred to as coefficients. For that particular predictor, the negative CI indicates that IADL among older adults was concentrated among poor older adults, and vice versa. The disadvantaged group for IADL included older adults aged 70–79 years, women living with a spouse, employed older adults, and those who reported poor self-rated health. On the other hand, those who have completed secondary or higher education, are currently married, have children, suffer from chronic conditions, and reside in urban areas are more likely to focus. Furthermore, older adults’ educational status, household wealth quintiles, self-rated health, and location of residence were all important factors to IADL disparities among the aged. For example, education accounted for 67% of SES-related disparity among older adults, whereas household wealth accounted for 38.2% of SES-related inequality. Self-reported health and place of residence both had a significant role in IADL disparities among older adults, accounting for 5.6 and 2.2% of the overall disparity, respectively.

**Table 3:** Estimates of decomposition analysis for contribution of various explanatory variables for IADL among older adults in India

Background characteristics	Coefficient	Elasticity	Concentration Index	Absolute contribution	% Contribution	
**Age (years)**						
60–69	1.00					
70–79	0.62*	0.032	−0.015	0.000	1.3	
80+	1.63*	0.028	0.019	0.001	−1.4	−0.1
**Sex**						
Men	1.00					
Women	−0.55*	−0.063	−0.033	0.002	−5.7	−5.7
**Educational status**						
No education	1.00					
Below 5 years	−0.56*	−0.026	0.001	0.000	0.1	
6 to 10 years	− 0.83*	−0.045	0.260	−0.012	31.7	
11+ years	−1.58*	− 0.021	0.614	−0.013	35.2	67.0
**Marital status**						
Not in union	1.00					
Currently in union	−0.21*	−0.027	0.040	− 0.001	2.9	2.9
**Living arrangement**						
Alone	1.00					
With spouse	1.03*	0.029	−0.197	− 0.006	15.5	
With children	1.42*	0.191	0.089	0.017	−46.5	
Others	1.38*	0.021	0.092	0.002	−5.4	−36.5
**Working status**						
No	1.00					
Yes	−0.76*	−0.039	− 0.174	0.007	−18.8	
Retired	−0.21*	−0.001	0.518	−0.001	1.5	−17.3
**Having children**						
Yes	1.00					
No	0.03	0.001	−0.359	0.000	0.8	0.8
**Self-rated health**						
Good	1.00					
Poor	0.55*	0.054	−0.038	−0.002	5.6	5.6
**Chronic diseases**						
No	1.00					
Yes	0.14*	0.012	0.051	0.001	−1.6	−1.6
**Substance use**						
No	1.00					
Yes	−0.08	−0.004	−0.120	0.000	−1.3	−1.3
**Wealth quintile**						
Poorest	1.00					
Poorer	−0.13	−0.009	−0.338	0.003	−7.8	
Middle	−0.16	−0.010	0.138	−0.001	3.8	
Richer	−0.30*	−0.015	0.522	−0.008	21.0	
Richest	−0.33*	−0.010	0.761	−0.008	21.3	38.2
**Religion**						
Hindu	1.00					
Muslim	0.41*	0.005	0.146	0.001	−2.1	
Sikh	0.06	−0.001	0.311	0.000	1.2	
Others	−0.17	−0.002	0.295	−0.001	1.4	0.6
**Caste**						
Scheduled Caste	1.00					
Scheduled Tribe	0.06	0.000	−0.444	0.000	−0.5	
Other Backward Class	−0.05	0.002	−0.029	0.000	0.1	
Others	0.06	0.004	0.220	0.001	−2.5	−2.8
**Place of residence**						
Rural	1.00					
Urban	−0.16*	−0.003	0.248	−0.001	2.2	2.2
**State**						
Himachal Pradesh	1.00					
Punjab	0.05	−0.001	0.331	0.000	0.5	
West Bengal	0.56*	0.010	−0.163	−0.002	4.7	
Orissa	0.79*	0.015	−0.368	−0.006	15.4	
Maharashtra	−0.26*	−0.009	− 0.126	0.001	−3.2	
Kerala	−1.04*	−0.030	0.350	−0.011	28.9	
Tamil Nadu	0.22*	0.003	−0.221	−0.001	1.7	48.0
**Calculated CCI**				−0.037	100.0	
**Actual CCI**				−0.073		
**Residual**				−0.036		

*CCI* Concentration Index; *if *p* < 0.05

## Discussion

The current study examined functional disability along with impairments among older adults in India. Functional disability was measured with ADL and IADL, whereas impairments among older adults were measured with six reported impairments: hearing, vision, walking, chewing, speaking, and memory. To our knowledge, this is the first research to evaluate ADL and IADL, along with various impairments among older adults in India. We proposed decomposing socio-economic factors for all three measurable outcomes, i.e., ADL, IADL, and impairments. However, we ended up decomposing IADL only as the other two outcomes (ADL and impairments) had negligible socio-economic differences when calculated with the concentration curve. The results observed that nearly 7.5% of older adults were not fully independent for ADL. More than half of the older adults (56.8%) were not fully independent for IADL, and nearly three-fourths of the older adults (72.6%) reported impairments. The overall prevalence of ADL and IADL among older adults in India was higher than the prevalence of ADL and IADL among older adults in China [30] and in the United States [31].

### Activities of daily living among older adults

In this study, ADL was significantly higher in older adults aged 80+ years, currently living with children, currently not working, having poor self-rated health, and chronic diseases. The findings are in concordance with the previous studies in the Indian setting [9]. Increasing age is one of the most significant variables in the study of ADL. Previous studies have unanimously highlighted that as age increases, people tend to observe a lower score on ADL means they are more likely to face functional disability related to ADL [32]. In general, we observed a higher percentage of older women who were not fully independent for ADL than older men. However, we could not find the significance of this result in our logistic regression model. However, various previous studies have significantly earmarked that older women tend to have a higher functional disability than older men [9, 14]. Women in India tend to ignore their health and generally avoid seeking health care, which may further cause poor functional disability [33].

The study noted that working older adults were less likely to report problems associated with ADL than non-working older adults. Previous studies are in line with this study in finding that older adults who work were less likely to report issues on ADL than their counterparts [34]. Working protects older adults and acts as a safety net against reducing activities of daily living [34]. Working older adults have to travel every day in quest of their work, and hence they are less likely to report poor scores on activities of daily living. Self-rated health and chronic diseases were also found to be significant crusaders for ADL among older adults. Poor self-rated health and older adults with chronic diseases were more likely to report issues with ADL than their counterparts. Studies unanimously highlighted that chronic diseases and poor self-rated health are the two most significant variables in the ADL study [9, 35]. Studies have noted that older adults tend to suffer from various chronic diseases, resulting in functional disability among them [36]. A study is of the opinion that chronic disease is the most important factor affecting ADL among older adults [16].

### Instrumental activities of daily living among older adults

As the age of the older adult increases, functional disability related to IADL increases among older adults. This finding is in concordance with previous studies, where a positive association was observed between poor responses on IADL and the increasing age of the older adults in various settings [37, 38]. Results noticed that women older adults had a better outcome on IADL than men older adults; this means men older adults tend to have poor IADL than women older adults. Studies have mixed responses to this finding as some studies noted that women older adults have higher levels of health-related limitations of IADL than men older adults [39]. In contrast, some studies concordance with this study in finding that men older adults have greater levels of health-related limitations of IADL than women older adults [38, 40]. A study noted that men were more likely to report needing help with cooking meals, doing laundry, and taking medicines, and this has substantial weightage on why a higher percentage of older men report limitations with IADL than older women [41].

This study has found that an increase in education decreases the IADL related limitations among older adults. Older adults with 11+ years of education were around 80% less likely to report limitations for IADL than older adults who had no education. Previous literature also highlighted the importance of educational attainment in decreasing the likelihood of reporting limitations for IADL among older adults [42]. Hu et al. (2005) believe that increased resource availability associated with higher education may improve self-perception and decrease limitations with various health conditions [42]. The study noticed that older adults living alone had lower odds of limitations related to IADL than older adults living with a spouse or with children or with anyone. This finding implicates that older adults living alone tend to help themselves by carrying out work required for daily living; thus, they are less likely to report limitations with IADL than their counterparts. Also, it might be an inference that the elderly living alone does not have any social support, and therefore they had to carry out the work on their own, improving their score on the IADL scale. Francisco et al. (2018) also noticed that older adults who live alone tend to achieve better outcomes on activities related to IADL [43].

Results highlighted that working older adults had lower odds of reporting poor IADL than non-working older adults. Previous studies agree with this study in noticing differences in IADL with working status [40]. It can be understood that working older adults may tend to be physically active, which is why they report better outcomes for IADL. Studies have noted that physical activities improve IADL among older adults [44]. Poor self-rated health and chronic diseases among older adults were linked with a poor score on IADL. Previous studies also highlighted that chronic disease and poor self-rated health affect limitations related to IADL among older adults [38, 43]. Regarding the possible relationship between SRH and IADL, Tomioka, Karumatani, & Hosoi (2017) believe that older adults with better SRH may be more likely to engage in social activities that promote better outcomes for IADL among them [38].

This study also examined socio-economic inequality in the prevalence of IADL among older adults in India. Results noticed that educational status and wealth quintile explained most of the socio-economic inequality in the prevalence of IADL among older adults. Previous studies also highlighted the importance of wealth in reducing inactivity related to IADL among older adults [44]. Income provides access to older adults to modify their current living conditions, positively affecting IADL [45].

### Impairments among older adults

This study also examined various impairments (hearing, vision, walking, chewing, speaking, and memory) among older adults, along with examining ADL and IADL. Results noticed that impairments were higher among older adults aged 80+ years, older adults with poor self-rated health and suffering from chronic diseases, older adults indulged in substance use. The impairments were lower among working older adults and older adults who were currently in a union. Previous studies unanimously highlighted that as age increases, older adults tend to perform poorly with hearing, vision, and other impairments [46]. A study has noticed that age-related hearing loss was the third most prevalent chronic medical issue among older adults [47]. As age progresses, a study has highlighted that memory hampers among older adults, inadvertently not associated with education level [48].

### Strengths and limitations of the study

One of this study’s main limitations is the self-reporting of data related to ADL, IADL, and impairments. Previous studies also assessed these measures as per the self-reporting of the respondents [14]. Furthermore, the study used chronic disease and self-rated health as two independent variables. These measures were also self-reported and may have biasness. Previous studies also used a self-reported measure of chronic disease and self-rated health as measuring chronic disease clinically may not be feasible [14, 49]. Furthermore, due to the cross-sectional study, we could not identify a causal relationship. Despite various limitations, this study has quite a few strengths that make this study unique. At first, this study examined various factors associated with impairments along with ADL and IADL, thus, adding one extra dimension to the study related to ADL and IADL. Moreover, the study intends to decompose the factors associated with inequality in the prevalence of ADL, IADL, and impairments; however, ended up decomposing only IADL as the other two factors were not having any significant observed socio-economic inequality.

## Conclusion

This study examined factors associated with functional disability and impairments among older adults in India. The study also intends to examine the contribution of various socioeconomic inequality factors in ADL, IADL, and impairments but ended up examining socio-economic inequality for IADL only as the other two factors did not show considerable socio-economic differences as measured through the concentration curve. It is proposed that while recommending any policy for older adults, it is important to consider the age and gender of the older adults and the current living status of the older adults. It is recommended that the government advise older adults to adopt health-promoting approaches, which may be helpful.

It is imperative to limit functional dependence among the elderly by providing adequate care to them. The government shall focus on providing comprehensive care to early older adults to check the functional limitation at the later age among them. Effective implementation of the National Program for the Health Care of Elderly (NPHCE) might provide an opportunity for improved health among the elderly. It provides promotional, preventive, curative, and rehabilitative services in an integrated manner for the elderly in government health facilities. It is recommended to implement the National Program for Health Care of the Elderly (NPHCE) by setting up geriatric clinics at Primary Health Centre. The setting up of geriatrics clinics will provide curative services to the older adults and prove to be a milestone in fulfilling the healthcare needs of the elderly, keeping in mind the ever-increasing older adult population in the country [17].

This study has demonstrated a substantial burden of chronic diseases on ADL, IADL, and impairments among older adults, further supporting the public health relevance of multi-morbidity among older adults. Therefore, there is a pressing need to deliver quality care to older adults suffering from chronic conditions.

## Supplementary Information

**Additional file 1.**

## Data Availability

“The data cannot be shared publicly as it is collected and stored by Institute for Social and Economic Change, Bengaluru, Karnataka, India (http://www.isec.ac.in/). However, other researchers may send data access requests to the director of the institute at director@isec.ac.in.” or at india.office@unfpa.org

## Abbreviations

ADL: Activities of Daily Living
BKPAI: Building a Knowledge Base on Population Aging in India
CI: Concentration Index
CI: Confidence Interval
IADL: Instrumental Activities of Daily Living
IEG: Institute for Economic Growth
ISEC: Institute for Social and Economic Change
NPHCE: National Program for the Health Care of Elderly
OBC: Other Backward Class
AOR: Adjusted Odds Ratio
PSU: Primary Sampling Unit
SC: Scheduled Caste
SES: Socio-economic Status
ST: Scheduled Tribe
TISS: Tata Institute for Social Sciences
UNFPA: United Nations Population Fund
