# Supplementary material for: Socio-economic inequality in functional disability and impairments with focus on instrumental activity of daily living: a study on older adults in India
Source: BMC Public Health. 2021 Aug 12;21:1541. doi: 10.1186/s12889-021-11591-1 (PMC8359266; doi:10.1186/s12889-021-11591-1)
Supplement: Supplementary file 1 — Additional file 1. [file 12889_2021_11591_MOESM1_ESM.docx]

**Supplementary table A:**

**1. Formation of Index of ADL:**

**Type of ADL** (0 “no” and 1 “yes”)

1. Bathing
2. Dressing
3. Toilet
4. Mobility
5. Continence
6. Feeding

The response were: 1. Do not require assistance…0

2. Required partial assistance…1

3. Required full assistance…1

Now I am going to ask you some questions regarding instrumental activities of daily living (IADLs) which are activities that are not necessary for basic functioning of daily life, but they let an individual live independently in a community.

The first category ‘do not require assistance’ depicts full independence and the remaining two categories were combined together and depict ‘not fully independent.’ The score of 0-6 was then generated using egen command in STATA 14. A score of 6 was categorized as 0, which represents full independence, and five and less were categorized as 1, which represents not fully independent to do activities of daily living.

---------------------------------------------------------------------------------------------------------

**A.2: Type of IADL** (0 “no” and 1 “yes”)

1. Ability to use telephone

Operates phone on own initiative----0

Dials a few well known numbers-----0

Answers the phone but does not dial--1

Cannot use phone------------------------1

2. Shopping

Takes care of all shopping needs independently--------0

Shops independently for small purchases----------------1

Needs to be accompanied on any shopping trip---------1

Completely unable to shop---------------------------------1

3. Food preparation

Plans, prepares and serves adequate meals independently---0

Prepares adequately means if supplied with ingredients------1

Heats, serves meals; does not maintain adequate diet---------1

Needs to have meals prepared and served----------------------1

4. Housekeeping

Maintains house alone or with help for heavy work----------0

Performs light daily tasks e.g. dish washing, bed making----0

Performs light daily tasks but cannot maintain cleanliness----0

Needs help with all home maintenance tasks---------------------0

Does not participate in any housekeeping tasks------------------1

5. Laundry

Does personal laundry completely ................ 0

Launders small items, rinses socks, etc. ...............0

All laundry must be done by others ............... 1

6. Transportation

Travels independently on public transport/own car ............... 0

Travels on public transport when accompanied by others ............... 0

Travel limited to car with assistance from another person ............... 1

Does not travel at all ............... 1

7. Medication

Is capable of taking medicines in correct dosage at correct time .......... 0

Takes medicine if given in separate dosage .................. 1

Is not capable of dispensing own medicines ..................... 1

8. Finances

Manages financial matters independently (budget, cheques, bills) ......... 0

Manages day to day purchases, but need help with banking, etc. ............. 1

Incapable of handling money ............ 1

The score of 0-8 was then generated using egen command in STATA 14. A score of 6+ was categorized as 0 representing full independence for IADL and score of 5, and less was recoded as 1 representing not fully independent for IADL.

----------------------------------------------------------------------------------------------------------------

**A.3: Impairment (Do you have any of the following difficulties?) (0 “no” and 1 “yes”)**

1. Vision

2. Hearing

3. Walking

4. Teeth (chewing)

5. Speaking

6. Memory

The responses were:-

1. Yes, fully……….1

2. Yes, partially……….1

3. No……… 0

The responses were then recoded as 0 “no impairment” and 1 “any one impairment”.

**----------------------------------------------------------------------------------------------------------**
